# Supplementary material for: Are carbon emissions trading and green financial instruments synergistic? -Comprehensive quantitative research based on content analysis
Source: PLoS One. 2024 Mar 7;19(3):e0298601. doi: 10.1371/journal.pone.0298601 (PMC10919601; doi:10.1371/journal.pone.0298601)
Supplement: S1 Data — (ZIP) [file pone.0298601.s001.zip › Data/地级市Z_F工作报告.zip!/║┌┴·╜¡/║╫╕┌╩╨/└┤╫╘╣1⁄2╓┌║┼í╛┬φ┐╦╩2╛▌═°í┐_2006.docx]

各位代表：

我代表鹤岗市人民政府向大会作工作报告，请予审议。

一、2005年工作回顾

 过去的一年，我们在省委、省政府和市委的领导下，坚持以经济建设为中心，以科学发展观为指导，突出重点，创新措施，真抓实干，合力攻坚，圆满完成了市十三届人大五次会议确定的各项任务，全面完成了“十五”计划。

——经济持续快速发展。地区生产总值达到113.7亿元，比上年增长13.4%。其中，第一产业实现增加值26.5亿元,增长2.2%；第二产业实现增加值47.7亿元，增长21.8%；第三产业实现增加值39.46亿元，增长12.6%。财政收入完成13.67亿元，增长63.7%。全社会固定资产投资完成32.36亿元，增长22.3%。

——结构调整步伐加快。工业的主导作用更加突出。实现增加值33.5亿元，增长21.5%，地方规模以上工业实现利税1.31亿元，增长66.3%；区域工业占整体经济的比重达到39%，比上年提高6个百分点；能源工业和食品工业规模扩大，非煤产业占工业的比重达到29.7%。农业和农村经济继续加快发展。粮食总产量达到9.2亿斤，增长6.6%；“主辅换位”加速推进，牧业增加值达到2.4亿元，增长23.2%。第三产业健康发展。社会消费品零售总额达到28.6亿元，增长10.4%；旅游业有新发展，共接待国内外游客37万人次，实现旅游收入3315万元，增长11.6%。民营经济进一步壮大。实现增加值21.8亿元，增长19.3%，实现税收1.5亿元，增长78.1%。

——项目建设成果丰硕。把项目建设作为经济发展的主要载体，开展连续三年“项目建设年”和“看项目、找差距、促发展”活动，实行了领导包项目、全员全社会全区域抓项目的推进机制，项目建设成果丰硕。全市共实施521个项目，投资千万元以上项目35个、亿元以上9个，形成了开工建设一批、签约落实一批、招商洽谈一批、谋划储备一批的格局。

 ——城乡建设稳步推进。城市住宅建设加快。开工63万平方米，竣工55万平方米，沉陷区治理二期工程开工建设61万平方米。基础设施建设进展顺利。绥滨至名山公路开工建设，绥滨至普阳、鹤北支线公路已完工；新建通村公路9条，修建了4条城市干道和19条街巷道路；投资1000余万元，完成了小鹤立河护岸、梧桐河防洪治涝、城区防洪等重点水利工程。城市绿化、亮化、硬化、净化又有新提高。义务植树30.4万株，专业植树1.69万株；在12条道路安装路灯122基；完成1.2万平方米硬化任务，城市面貌又有新变化。

 ——改革开放不断深入。各项改革取得重要进展。企业改革深入推进，企业职工“并轨”基本完成，粮企改革完成阶段性任务。教育改革稳步实施，全面完成了中小学人事制度改革、企业分离办教育体制改革。农村信用社改革基本完成。对外经贸合作向纵深发展。共完成7800公顷土地种植、5000平方米房地产开发等5个对俄经贸合作项目；对外贸易大幅度增长，进出口总额完成1415万美元，增长87%；利用外资实现168万美元，增长两倍；口岸运行态势良好，出入境货物20.13万吨，增长18%，过客1.7万人次；口岸过货量位居全省第五位。招商引资取得新突破。到位资金45.3亿元，完成年目标的113.3%，国内招商引资项目资金19.39亿元，比上年增长98.7%。

 ——社会事业全面进步。科技进步取得新成效。争取省级科技项目立项12项，获取专项资金支持331.6万元；加强了与科研院所和大专院校的产学研联合。教育事业加快发展。重点改善了9所学校的办学条件；启动了教育资源三年整合规划，实施了新一轮中小学教师培训工程；教育教学质量稳步提高，高考升学率达到93%，中等职业教育在校生占高中阶段教育的比例达到23%，高等教育就业率达到60%以上。卫生事业得到加强。投资1100万元建设了传染病院，投资400万元的急救中心投入使用，医疗救治体系进一步完善；社区卫生服务网络基本形成，卫生服务站覆盖面达100%；农村合作医疗试点工作进展顺利，农民参合率达62%；爱国卫生运动深入开展，各类创建工作全面达到省级标准。文化事业繁荣活跃。整合文化资源，组建了市艺术剧院，增强了专业艺术团体的活力；群众文化活动丰富多采，参加省级以上比赛获52项奖励，组织“纪念建市60周年”等大型专场文艺演出221场。体育事业蓬勃发展。全民健身运动日益普及，竞技体育有新提高，参加省级以上各类比赛共获金牌48枚。广播电视工作实现新突破。新增有线电视光缆300多公里，结束了多路微波传输和租用光缆的历史；新开4个有线电视频道，数字电视开始试播。计划生育保持全省先进水平。人口出生率2.57‰，自然增长率0.29‰，符合政策生育率97.7%。信访工作得到加强。解决了一大批处理难度大的信访积案，进京进省集体访下降60%和76.5%，个人访下降49.5%、34.5%。“平安鹤岗”建设成效显著。刑事案件发案下降，破案率提高，没有发生涉枪、涉暴、绑架等严重暴力案件；命案发案下降35.4%，破案率92.7%，比公安部标准提高7.9个百分点，命案必防经验在全省推广和交流；入室盗窃、抢劫案件下降31.9%、24.1%，人民群众安全感明显增强，社会治安创近年来最好水平。安全工作全面加强。全市各类事故下降19%，事故死亡首次降到百人以下，比上年减少34人，比省下达控制指标少25人，综合安全和煤矿安全工作均列全省第一位，连续三年受到省政府表彰、重奖；煤矿安全生产保持了稳定态势，百万吨死亡率1.98，比上年下降0.86，鹤矿集团百万吨死亡率0.98，下降1.16，市、矿均没有3人以上死亡事故，实现了市、矿平安；消防连续10年未发生重特大火灾，交通、森防、非煤矿山、建筑、危险化学品和特种设备等各项安全形势进一步好转。环保工作继续加强。对新建项目严格把关,强化了对污染源的治理,整改了放射源隐患,加大了生态保护和水源保护力度，达到了省核定控制标准。外事、侨务、民族、宗教、民政、档案、司法、国防、人防、双拥、人事、物价、统计、地震、残联等工作均取得了可喜成绩。

 ——就业解困成效显著。社会保障功能不断增强。实现就业和再就业27560人，城镇登记失业率控制在4.3%以内；养老保险金、失业保险金实现了按时足额发放；城市低保应保尽保，人均补差标准提高5元。困难群体得到更多关注。沉陷区治理二期工程辟建2个小区113栋楼房、61万平方米。筹集资金240万元，为受灾群众新建、维修房屋1123间；发放款物379万元，救助困难群众39065人；发放助学资金42.6万元，资助困难学生4185人。补还历史欠帐力度加大。筹集资金7240万元，补发拖欠职工工资、医药费，偿还政府拖欠工程款、城市基础设施、公益事业建设负债等。

 ——人民生活明显改善。城乡居民收入大幅度增长。城镇人均可支配收入达到7489元，增长14.6%；农民人均纯收入3468元，增长9.2%。城乡居民储蓄存款余额119亿元，比年初增长8%。人民生活质量持续提高。城市人均住宅建筑面积21.5平方米，农村21.71平方米，分别增加2.7平方米、0.37平方米。适度调整热价的同时，将热费补贴标准提高了20%；在全省率先出台了新的城市拆迁补充规定，调整了城市拆迁政策，提高了拆迁补偿标准，保障了被拆迁群众的利益，加快了拆迁进度；投入140万元新增20套社区居民健身器材。

 ——工作机制更加完善。依法行政深入实施。深化了行政审批制度改革，取消行政许可事项115项。阳光政务加快推行。实行了公开办事和公示制度，城市建设配套费统一标准不再实行弹性减免和行政收费项目与标准等重大事项以公告、通告的形式向社会公开，接受监督。科学决策、民主决策水平有新提高。实行了重大决策听证、论证制度，就重大信访案件、小公共汽车经营权出让、城市建设工程拆迁、社会保障体系建设、公共卫生管理、热费和公交车票价格调整等重大问题举行座谈、听证、论证会63次。社会监督进一步加强。实行了部门述职评议制度，在47个行政执法、经济管理和社会服务部门中开展了“端正行风，促进经济发展”最佳最差单位评议活动，城管、财政、交通3个职能部门接受了市人大的述职评议。便民服务有新进展。组建了“人民办事中心”，将涉民办事项目尽力纳入中心，实行了真正意义上一站式办公、一条龙服务、中心内受理、中心内办结，固定了收费项目、收费标准、办事程序、办事时限，较好解决了办事难、办事效率低和弄权勒卡等弊端。中心启动以来共受理业务2.98万件，办结率97%，人民满意率98%，方便了外来投资者和人民群众，优化了经济发展环境。与人民群众的沟通联系不断扩大。实行了政府新闻发言人制度，就全市经济社会发展等情况组织召开了新闻发布会，扩大了人民群众的知情权和参与权。政府服务效率和水平有新提高。实行了分级协调服务制度，共协调处理各类重点问题61项。工作运行管理机制进一步完善。实行了全员目标责任管理机制，进一步解决了机关工作的“大锅饭”问题，提高了工作效率。改善发展环境力度加大。实行了执法执收执罚人员专项管理制度，对违纪行为制定了严格的处罚办法，加大力度治理了影响经济发展的痼疾顽症。强化了工作落实的督查督办。建立了督办督查与责任追究制度，加强了对政府决策、重点工作落实情况的监督检查，促进了工作落实。强化和完善了激励机制。设立了专项资金，对拔尖人才、招商引资、纳税贡献大户、项目投资和目标考核达标优胜单位进行奖励，激励全市人民干事创业、建功立业。

 各位代表，过去的一年，我们众志成城，战胜了超百年一遇的特大洪水；积极应对，夺得了防控松花江水体污染的胜利；以积极节俭、昂扬进取的方式纪念了建市60周年；全市人民以经济建设为中心，合力攻坚，强力推进，坚持科学、协调、可持续发展，取得了经济快速发展、社会事业全面进步、项目建设成效显著、全市安全稳定、人民生活进一步提高的丰硕成果。这些成绩的取得是省委、省政府和市委正确领导的结果，是市人大、政协大力支持、依法监督、民主监督的结果，是全党抓经济、全民创业、全员招商、全社会抓项目、全区域抓发展、合力攻坚的结果，是全市各级组织、广大干部职工和人民群众共同奋斗的结果，是中省直部门和社会各界大力支持的结果。在此，我代表市政府向全市人民和各级干部，向驻鹤部队、武警官兵和公安司法干警，向贡献于鹤岗发展的市内外投资者，向关心和支持鹤岗经济建设和发展的社会各界人士，向对政府工作给予大支持和帮助的市人大、政协、各民主党派、人民团体表示崇高的敬意和衷心的感谢！

 全面总结过去一年的工作，我们还必须清醒地看到经济社会发展和工作中存在的问题和困难。经济发展缓慢，经济总量小，总体实力弱仍然是主要矛盾；结构性矛盾突出，资源精深加工产业、科技含量高的新兴产业和民营经济弱小仍然没有大的改变；企业效益质量低、粗放经营的问题仍然没有根本解决；项目建设刚刚起步，差距大、困难大、风险大，任务仍然繁重艰巨；历史积淀遗留的问题多，补还历史欠帐、保障救助民生民困、维护社会稳定、实现生产安全的困难和压力仍然巨大；政府自身建设仍然有很多问题不尽人意。这些问题我们将在今后的工作中认真加以解决。

二、今后五年的总体构想

 今后五年是我市实施“十一五”规划，实现老工业基地振兴的关键时期，是推进经济快发展、大发展、跨越发展、科学发展，构建和谐社会的重要历史阶段。

 面对新的形势，“十一五”期间经济社会发展的指导思想是：以邓小平理论和“三个代表”重要思想为指导，全面贯彻党的十六届五中全会、省委九届七次全会和市委九届十一次全会精神，坚持以科学发展观统领经济社会发展全局，在经济工作中，坚持工业立市为主导战略，资源精深加工为主攻方向，项目建设为主要载体，招商引资和全民创业为主体措施，环境优化、人才开发和基础设施建设为主力保障，加速建设两大基地，发展六大产业，迅速扩张经济规模和经济总量，努力提高经济效益和经济质量，大幅度增加财政收入；加快资源型城市转型，发展接续替代产业，调整经济结构，转变经济增长方式，增强长远竞争力；全面发展社会事业，提高人民生活水平，健全公共服务体系，保护资源环境，构建和谐平安鹤岗，为全面建设小康社会奠定更加坚实的基础。

 依据上述指导思想，“十一五”期间经济社会发展的主要目标是：到2010年，地区生产总值达到200亿元，年均增长12%；财政收入达到20.8亿元，按可比口径年均增长12%；固定资产投资累计达到332亿元，年均增长25%。外贸进出口总额3820万美元，年均增长22%；五年实际利用外资1500万美元，年均增长8.6%；城镇居民人均可支配收入达到12000元，年均增长10%；农民人均纯收入达到5090元，年均增长8%；社会消费品零售总额达到48亿元，年均增长11%；资源利用率比2005年提高20%，能源消耗率下降20%。已经规划的资源精深加工、接续替代产业的重点项目基本建成，“两大基地、六大产业”的经济格局基本形成，构建起较大范围控制资源、占有市场、具有较高效益和较强竞争力的主导产业和接续替代产业体系；城市功能明显增强，棚户区改造、城市新区开发建设和城乡道路、水利工程、公共设施建设、各项社会事业发展取得更大成效，人民生活明显改善，构建和谐社会取得重大进展，整体经济走上又快又好的可持续的科学发展道路。

 三、2006年工作任务

 今年是实施“十一五”规划的开局之年，是乘势而上、加快发展的关键一年。根据市委九届十一次全会精神，今年工作的总体要求是：认真贯彻中央和省的各项重大部署，坚持以科学发展观为统领，以推进老工业基地振兴和资源型城市转型为主线，围绕增加财政收入、富裕人民群众、构建和谐社会，抓经济、搞建设、谋发展。强力推进项目建设、招商引资和优化环境工作，节约资源，保护环境，转变增长方式，实现又快又好发展。稳定煤炭产量，加强煤矿安全，着力抓好煤炭深加工和农产品深加工等各类工业项目，支持现有各类企业加快发展；发展绿色特色农业，增加水稻面积，扩大畜牧规模，发展林业经济，建设社会主义新农村；壮大旅游、商服、金融、通信等第三产业；推动对俄经贸合作战略升级；搞好城市建设，改善人居环境。加强人才工作，鼓励干事创业。抓好就业、社保、科技、教育、卫生、文化、体育等各项社会事业，做好困难群众的帮扶救助工作。加强精神文明和民主法治建设，实现“三个文明”协调发展。全面加强政府自身建设，努力开创各项工作新局面。今年全市经济社会发展的预期目标是：地区生产总值增长12%以上；财政收入按可比口径增长10%以上；固定资产投资增长25%以上；社会消费品零售总额增长10%；城镇居民人均可支配收入增长9%；农民人均纯收入增长8%。

 为确保预期目标的实现，我们要集中力量努力做好十二项重点工作。

 (一)以项目建设为重点，推进工业结构、质量战略升级。

 今年是全市三年“项目建设年”的第二年，是攻坚之年，要以项目建设为载体，继续坚持去年好的思路和机制，进一步硬化措施，加大力度，强力推进，取得更大成效，带动工业经济优化结构、扩大规模、提高质量效益。

 抓好项目建设。围绕“两大基地、六大产业”规划的项目，加快推进和落实。要抓好在建项目建设。对已开工的60万千瓦电厂二期工程、120万吨鸟山煤矿、10万千瓦矸石电厂、90万吨焦炭、8万吨合成氨13万吨尿素、5万吨玉米酒精等10个项目要加强组织和服务，加快建设速度，使其尽早投产达产。要抓好已签约和招商项目的推进。对4.3万吨焦油、120万吨甲醇、100万吨化肥、10万吨酒精、三精千鹤等10个项目要跟踪落实，促其尽快开工建设。要抓好未落实项目的招商。下大力气招商引资和发动民营企业投资，对林木加工、新型建材、煤层气开发、肉牛加工等项目尽早落实投资者。要积极谋划有市场、有优势的新项目。立足自身优势、适应市场需求、跟紧科技进步，积极谋划200万千瓦电厂三期、180万千瓦龙江电站等一批有规模、有质量、有效益、有科技含量、有竞争力的新项目、好项目，做好论证、报批等前期工作，充实优化项目库。要抓好现有企业的技术改造和扩产。对市场前景好、经济效益高、社会贡献大、有发展潜力的现有企业和项目加大扶持服务力度，加速扩产改造，促其做大做强。

 规划建设工业园区。遵循经济发展规律，从我市发展需要出发，规划建设工业园区，实现聚集发展、规模开发、集中建设、统一服务。总体规划建设三个园区，一是规划建设以新华电厂一、二、三期、煤焦化、化肥、甲醇、煤层气、矸石电厂等项目为主体的新华煤电化工业园区。二是完善扩大鹤西高新技术产业工业园区，以现有的系列医药生产企业和万禾源油脂、鹤鸣米业等食品企业为基础，辟建成高新技术产业工业园区。三是规划建设宝泉岭龙头企业工业园区，以大豆、生猪、乳品、粮食精深加工等龙头企业为重点，建设大型龙头企业工业园区，带动农业产业化经济快速发展。今年要投资3000万元，进行基础设施建设；组建园区机构，加强园区管理。

 创建宽松环境。加大力度解决影响经济发展环境的重点难点问题，为项目建设和经济社会加快发展提供保障。努力优化执法环境。把严格执法与为经济发展、项目建设服务有机结合起来，严肃查处执法谋私、弄权勒卡、刁难企业、障碍发展的行为，保护企业和人民群众的合法权益。切实提高政府服务水平。进一步放宽政策，优惠企业，转变职能，优质服务。办好人民办事中心，发挥优质服务、高效办事、优化环境主阵地的作用；组建项目建设服务中心，逐步为企业提供信息、项目、技术、人才、项目论证报批等系列化服务；把担保中心办成独立法人实体，扩大资本金，提高担保融资能力，着力解决中小企业贷款难的问题；管理好开源国有资产经营公司，搞好城市经营、国有资产运营和基础项目建设经营，成为促发展、有效益、有实力的实体。

 (二)以建设新农村为重点，推进农村经济社会发展战略升级。

 要抢抓建设新农村的历史机遇，努力争取国家和省的政策、资金支持，增加财政投入，组织农民积极参与，加快水利、道路等农业、农村基础设施建设，推进教育、卫生、文化、体育、环保等社会事业发展，促进农村经济发展和农民收入增加。以生产发展、生活宽裕、乡风文明、村容整洁、管理民主为目标，实现新农村建设开好局、起好步。

 稳定发展粮食生产。调整种植结构，突出发展水稻，推进江萝、德龙、团结三大灌区建设。引进先进适用技术，提高种植业集约化水平，实施标准化生产；加快土地使用权有序流转，逐步把土地向种田大户转移；提高农业机械化水平，更新、改造大中型农机具；发挥农垦生产力优势，扩大代耕代种、场县联合、跨区作业，实行农机、农技资源共享。粮食总产争取达到10亿斤。

 突出发展畜牧业。深入实施“小区大户”战略，大力推进“主辅换位”，提高科学饲养水平，完善畜牧社会化服务和防疫灭病体系，积极预防禽流感。奶牛存栏达到1.4万头，增长16%；肉牛12.1万头，增长10%；生猪65万头，增长16.1%；牧业增加值2.6亿元，增长16%；牧业人均纯收入1342元，增长10%。

 推进产业化经营。发挥龙头企业的带动作用，整合品牌，延长产业链；引进大的战略投资者建设高标准农牧资源精深加工企业；龙头企业要开拓市场，提高生产加工能力。完善、创新基地与龙头企业的利益联结机制。

 积极发展农村基础设施建设和公益事业。积极完成国家和省安排的农村基础设施和公益事业项目，设立市级新农村基础设施建设资金，引导乡村集体和农民资金，加快村屯基础设施建设。完成梧桐河防洪除涝、大鹤立河及小鹤立河堤防、元宝山水库续建和五号水库岁修等重点工程，做好小鹤立河水库除险加固、绥滨松花江大桥和关门嘴子水库前期工作。完成农村饮水安全工程20处，解决饮水村屯20个，人口2万人。建设康居工程10万平方米，完成267公里通村公路建设任务。抓好省级两镇21村的新农村试点工作，启动市里第一批规划的10镇 百村的新农村建设，在两县一区各建一个新农村建设样板示范村。加强农村卫生工作，完善农村医疗保障体系，抓好农村合作医疗；落实大病补助、教育“两免一补”等政策，发展农村医疗保险、养老保险、最低生活保障等公共事业。

 推进农村民主管理和乡风文明建设。加快农村民主法制建设，扩大基层民主，搞好村民自治，健全村务公开制度，开展普法教育，确保广大农民群众依法行使当家作主的权利。加强农村精神文明建设，加快发展农村文化事业，倡导健康文明的新风尚。

 (三)以发展现代服务业为重点，推进第三产业层次和水平战略升级。

 着力推进第三产业市场化、产业化和现代化进程，壮大服务经济，提升质量水平，发展新兴产业。

 把旅游培育成新兴产业。要适应国内外对生态旅游的旺盛需求，发挥我市山、水、平原、界江、边境等丰富的旅游资源优势，加快开发建设，努力把旅游业培育成新兴产业并做大做强。建设好现有旅游景区。建设市区至细鳞河国家森林公园道路，建设经营好细鳞河民俗风情园、松鹤西湖公园、将军石山庄、鹤北红松母树林公园，提高档次和水平。打造新的旅游热线。发挥我市黑龙江界江与哈市距离最近和龙江三峡的地缘资源优势，开发启动黑龙江界江游，同伊春联合启动五营森林公园、汤旺河兴安石林国家地质公园、嘉荫小兴安岭恐龙博物馆、龙江三峡等小兴安岭和黑龙江界江山水游，开通哈尔滨——伊春——嘉荫——黑龙江——名山岛——鹤岗的旅游热线。今年购置两条游船，开通黑龙江三峡界江游。要加快建设太平沟清代黄金文化游接待设施，高标准规划建设名山风景区，筹建黑龙江自然史馆，逐步建成黑龙江流域标志性、权威性的自然人文景观。要强化旅游产品宣传。加强旅游产品促销，加快旅游纪念品开发，扩大旅游品牌宣传，叫响“不到黑龙江不算到龙江、要到黑龙江最近最美是鹤岗”的旅游品牌，逐步形成在全国有特色、有竞争力的黑龙江界江游。

大力发展现代服务业。以培育农资、农贸、典当、旧物、二手车等要素市场为载体，推进商品市场体系、现代流通体系、市场监控体系和商业信用体系建设。加快商业业态重组，发展大超市，推进连锁经营，扩大物流配送。扶强扶壮骨干企业，积极培育大型商贸集团；鼓励连锁企业进社区，争取设立20个社区便民连锁店；深入实施“万村千乡”工程，争取设立150个连锁农家店。

 加快中介服务、金融保险、交通运输、通讯等产业的发展，推动第三产业上规模、上水平。

（四）以拓展领域和做大做强为重点，推进民营经济发展战略升级。

 民营经济是我市地方经济最具活力、最具发展潜力的主体力量，要通过政策引导和服务支持，促其做大做强、提档升级。

提升社会地位。我市民营经济不断发展壮大，对财政的贡献越来越大，吸纳就业越来越多，对经济增长的拉动越来越强，已成为地方经济的主体，必须高度重视，实行特殊的扶持政策，提供更加优质的服务，创建更加优良的环境。

拓展发展领域。近年来民营经济发展较快，特别是随着煤炭效益的持续好转，一些民营企业积累了较强的资金实力，具备了扩大投资、提升产业层次的条件。要宣传民营企业家热爱家乡、投资家乡、贡献家乡，让他们充分认识并坚信家乡有宽松的经济发展环境、诸多的人文社会资源优势，坚定在家乡发展的信心，优先选择在家乡投资兴业。要引导民营企业提升产业层次，调整投资结构，拓宽经营领域，扩大生产规模，在有资源优势、市场优势的“两大基地、六大产业”上建项目、办企业，发展资源精深加工业；鼓励民营企业参与城乡基础设施建设和公用事业经营，发展现代服务业。

优化政策和服务。实行内外投资同样优惠的公平政策，实现市内民营企业和招商引资企业一视同仁。从今年开始，开展评选有突出贡献民营企业家活动，对投资规模大、纳税水平高、吸纳就业多、贡献突出的民营企业家大力表彰奖励，在社会地位、就医就学等方面给予特别优待。强化资金扶持，尽力解决中小企业融资难的问题。对重点民营企业继续实行市、区领导联系与部门包扶等措施，及时帮助解决发展中的重点难点问题，使民营企业留得住、发展快、做得大。

 (五）以完善城市功能和城市新区开发为重点，推进城市建设管理战略升级。

 要以强化城市配套设施建设、完善城市功能、改造棚户区、启动城市新区建设为重点，进一步提高城市规划、建设、管理水平。

 搞好棚户区改造。按照省的规划部署，积极争取国家和省的支持，加大棚户区改造力度，按期完成改造任务。

 启动建设城市新区。根据城市建设发展的实际需要，按照城市建设总体规划，从拓展城市发展空间、增强城市承载功能、降低建设成本、提高规划建设水平、满足人民群众日益提高的对城市建设的需求出发，结合沉陷区治理和棚户区改造，启动建设城市新区。一是启动建设鹿林山城市新区，使城市建设同沉陷区治理配套，向南部工业园区、供水供热企业和兴安区靠拢，逐步建设分布小鹤立河两岸、环绕鹿林山自然公园的鹿林山城市新区。二是利用开行贷款平台，启动建设城市西部新区。两个城市新区要高起点规划，首先重点实施征地和主干道路、给排水、供热供电等基础设施建设，实现环境率的提升和新区土地升值，提高城市经营效益。

规划建设鹿林山城市自然公园。加快景点景区和基础设施建设，扩大绿化覆盖率，使其成为市民休闲娱乐、提高城市品位的最大城市自然公园。

加快沉陷区治理速度。完成沉陷区治理二期主体工程，加快C、D级危房搬迁和A、B级危房的维修加固。

 完善居民小区配套设施和功能。以加快居民小区配套设施和卫生、文化、体育、物业、社区等事业建设为重点，进一步完善居民小区社会功能，方便人民群众生产生活，提高生活质量。今年安排1000万元，修建50条街巷道路，新建改造30座公厕，新上270基路灯，实施小区绿化，改善卫生条件，建设商业网点。

加快住宅建设。实现开、竣工各50万平方米，建设6栋高层住宅。

扩大城市绿化。加快城区绿化步伐，筹资370万元，完成专业植树1万株，义务植树40万株，其中城内次干道、居民小区义务植树10万株。

继续搞好城市基础设施建设。筹集3000万元资金，开工建设和维修10条城市主次干道，完成绥滨至名山段公路拓宽改造和兴安北桥重建；改造供水管网和城市排水系统；加强城区防洪工程建设和综合治理；启动建设医疗废物处置中心；启动建设供热三期工程，争取新增供热能力100万平方米以上。继续实施“十小工程”，更多地倾斜于雪中送炭。

 加强城市管理。加强规划审批管理，杜绝私建滥建。加大依法拆迁力度，保证城市建设有序进行，依法维护拆迁双方和回迁户的合法权益。加强公共设施管理与维护，实施专项治理，建立长效机制，进一步提高管理水平。

(六)以扩大对俄经贸合作为重点，推进对外开放战略升级。

要以中国“俄罗斯”年活动为契机，努力扩大对俄经贸合作，扩大对周边国际市场的参与度，加强与国内发达地区的合作与交流，增强开发两个市场、利用两种资源加快发展的能力。

 扩大对俄开放。要扩大项目合作。在农业种植、畜牧养殖和农产品加工、森林采伐加工、矿产资源开发等领域做大做强。过境种植要达到1.2万公顷，扩大农副产品就地加工；林木采伐要达到15万立方米，年过境18万立方米，在境外进行部分成品和半成品加工；矿产资源开发要争取锰矿开采中标，黄金开采正式开工；房地产开发争取有新进展。要完善口岸功能。加快名山第二港区和报关报检大厅建设，加快口岸物流和对俄出口加工基地建设。新上一艘摆渡船，新建口岸货物仓库。口岸过货要达到22万吨，增长10%，过境2万人次。要强化协调、联系和服务。完善高层互访机制，恢复两州市经贸协调委员会制度，密切与俄方的沟通协调。组建“中俄商会”、“华人企业协会”，加强对赴俄企业的指导服务。

加强与国内经济发达地区的交流与合作。重视对国内发达地区的交往联系和经济合作，更多地引进、利用国内企业和项目、资金，提高“南联”水平。

 进一步参与国际经济分工合作。延伸发展触角，向俄远东地区和腹地、原独联体国家、东欧国家拓展。以优势资源和产品为依托，加强与东南亚、日韩、港澳台等国家和地区的合作，努力扩大产品出口，提高市域经济外向度。

 （七）强化招商引资主体措施，努力解决经济社会发展的瓶颈制约问题。

 要切实强化招商引资的主体措施，强力推进、扩大成效，努力突破资金、项目、人才、技术等项目建设、经济发展的瓶颈制约。招商引资到位资金要完成48亿元，其中市场招商36亿元，分别增长20%。

 进一步优化政策。完善出台新的招商引资优惠政策和项目投资者优待办法，在土地、收费、待遇等方面给予最大优惠，竭尽全力在煤电、煤化、食品加工、林木深加工、石墨、水泥生产加工等优势项目上引进大的战略投资者。要加大奖励力度，设立专项资金，重奖有突出贡献的单位和个人，鼓励、引导全民、全社会招商引资。

进一步扩大对外联系。主动出击，活跃对外交往。要更多地参加国家和省组织的各类招商洽谈活动；更多地组织各级领导、企业和有关专家走出去、谈项目、搞招商；有目的、有重点地在重点招商地区建立友好关系，设立联系机构，派专人常驻招商；更多地邀请战略投资者来我市考察、洽谈，增加招商引资联系机率和成功率。要整合区域内各大经济板块，深入发动全民全区域招商。

进一步加强领导。各级领导都要把招商引资、项目建设作为重点工作任务，强化领导作为和示范带动作用。市级领导要全部包项目、抓招商，政府副秘书长以上领导和县区政府主要领导要力争引进一个投资1000万元以上的大项目，市直部门主要领导力争引进一个投资500万元以上的项目，既为全市的招商引资、项目建设做出贡献，又为基层和全社会做出表率。此项工作年终作为专项指标进行评比和奖励。

 (八)强化财税金融工作，为经济社会发展提供强力支撑。

 要根据全市经济社会发展的需要，切实加强财税金融工作。

 强化财政功能。要更好地发挥财政资金的导向作用，调整支出结构，提高资金使用效益。加大对财源建设的支持力度，促进经济发展，安排3000万元用于承载项目的园区建设。支持新农村建设，安排500万元用于农村基础设施建设和农村公益事业发展。支持城市基础设施建设，增加3000万元用于城市建设，筹资1000万元用于城区防洪建设。支持城市社会事业发展，重点发展科技、教育、文化、卫生等事业，促进经济社会协调发展。支持公众利益和困难群体，安排4000万元用于改善民生、解决民困、维护民利的事业。支持社区建设，安排400万元，用于改善社区办公条件，提高社区工作人员待遇。支持历史遗留问题的解决，逐步偿还历史债务。支持各项重点工作，建立十项专项资金。支持部门强化职能、优质服务，补齐行政经费，增加公、检、法部门经费，支持这些部门和单位增加条件、强化职能、优质服务，增强抓经济发展的能力。要深化财政改革，继续实行“收支两条线”管理，完善政府采购和国库集中支付制度。创造条件，逐步实行部门预算制度。要坚持过紧日子的思想，搞好增收节支，严格控制一般性财政支出，加强财务审计和监督，严肃财经纪律，提高财政资金的使用效益。

强化税收征管。坚持依法治税，完善征管机制，提高征管水平，实现应收尽收；严厉打击偷逃漏税等违法行为，确保工商税收及时足额入库，保证财税任务的圆满完成。

 切实加强金融工作。经济社会的快速发展、项目的大规模建设既给金融工作提出了更加艰巨的任务，又为金融单位开拓市场、提高效益提供了空前机遇，必须切实加强金融工作。要加快社会信用体系建设，督促企业提高信用等级，建立良好的金融生态环境。积极筹措资金，加强对效益好、前景好、科技含量高、竞争力强的企业和项目的信贷支持。强化优质服务，创新金融运作机制。深化农村信用社改革，推进城联社改革。

（九）强化区域联合，努力形成互惠共赢、共同发展的合力。

 根据市场经济和区域经济发展的规律，从鹤岗板块经济的实际出发，加强联合合作，破除行政体制障碍，激活发展活力，实现区域内资源、政策和优势共享，促进共同发展。

 统一发展战略。把“建设两大基地、发展六大产业”作为全区域的共同战略，发挥各自优势，合力发展资源精深加工，培育接续替代产业。

 统一发展政策。把优势和发展共享政策覆盖全区域，对各经济单元的项目建设实行同样政策，促进全区域合力上项目、办企业、快发展。

 统一优质服务。进一步强化服务协调，支持鹤矿集团加快项目建设，做强煤炭主体产业，大力发展煤炭等资源精深加工项目、辅助改制企业创业上项目和利用闲置资产上项目。支持农垦系统加速推进农业产业化、现代化进程，支持利用国家扶持和自身实力大上农牧产品精深加工项目，增强对两县农业的带动服务功能。借助全省东部煤电化基地建设的机遇，支持鹤岗电厂二、三期工程建设，提高企业效益和对市域经济的贡献率。支持鹤北林业局加快林木深加工、过境采伐和旅游业发展。完善高层协调机制，定期研究解决共同发展中的重大问题。

 (十）强化资源节约和环境保护，努力建设资源节约型和环境友好型社会。

 落实科学发展观，坚持开发节约并重、节约优先的原则，努力以最小的资源消耗和环境代价获取最大的经济效益和社会效益，促进经济社会可持续发展。

 积极发展循环经济。重点发展粉煤灰和煤矸石、煤层气综合利用，搞好焦炉气综合利用和煤焦油深加工，发展粮食精深加工和综合利用，大力推进废物利用，使经济发展由线形运行向循环运行转变，逐步提高资源利用率。

 搞好资源节约。完善和推行行业节能、节水、节材、节地，切实抓好降低能耗工作，重点行业和重点企业能源消耗下降5.0%，公共建筑和新建城镇住宅执行节能50.0%的标准，单位生产总值能源消耗有大幅度降低。项目建设要优先盘活闲置资产。要在全社会大力提倡勤俭节约的好风气，节约食品和生活用品。

 加强生态保护。扎实推进生态市建设，开展建立黑龙江绥滨段岛屿湿地自然保护区的前期综合考察、申报工作；抓好五道岗生态示范区畜禽养殖污染防治试点工作，推进畜禽养殖污染防治；积极争取生态省建设专项资金支持，使生态市重点建设项目得到落实。加快封山育林、植树造林，继续搞好退耕还林、还草、还湿，加强水土流失治理。严格把好环境准入关，杜绝新污染源的产生。

 强化环境综合整治。深入开展城市大气整治，重点抓好城市集中供热，城乡推广使用洁净能源，扩大城市燃气、农村沼气覆盖面。继续实施烟尘污染、噪声污染治理工程，抓好省级宁静小区、无烟小区、环保锅炉房、模范工地创建工作。加强污染控制，对全市重点污染源实施排污许可，进行总量核定；强化放射源及医疗废物、危险化学品的日常监管，杜绝污染事故发生。搞好沉陷区综合治理，整治废弃矿山，积极争取污水、垃圾处理项目。

 (十一)推动社会事业全面进步，实现经济社会协调发展。

 优先发展教育。巩固基础教育，改善薄弱学校，加强学校危房改造，力争消灭D级危房；扩大优质教育资源，合并鹤岗一中和原鹤矿集团高中，创办国家级示范高中。大力发展职业教育，增加适用专业，扩大招生规模，启动实施技能型人才培养、农村适用人才培养、劳动力转移培训等五大工程。提升高等教育，采取与名校挂靠办学、联合办学，实施定向培养等方式，提高办学层次；落实好全省“乡村教师培养计划”；做好2007年国家复检工作。

 加快科技创新。继续增加科技投入，加快科技项目建设，选择一批科技项目争取在国家和省立项。进一步加强科技中介服务机构建设，更多地培育科技型企业。加快实施“企业信息化工程”、“农村信息化工程”和“城市地理信息化工程”，加快数字鹤岗建设。积极引进科技成果，提高自主创新能力，提高产业层次和水平。

 完善公共卫生体系建设。加强疾病预防控制，完善启用传染病院，加强乡镇卫生院建设，健全急救中心，提高处置突发性公共卫生事件的能力。

 推进文化事业发展。实施“文化信息资源共享工程”，改造影剧院等文化设施。增加农村村屯和城市小区的文化设施，深化专业文艺团体改革，激发活力，强化功能。

 加快发展广播电视事业。加快有线电视光缆改造进度，新增数字电视有线频道30套，新增有线电视用户5000户；省电台3套节目落地开通。坚持资源共享原则，建设广播电视演播大厅。

 加快体育事业发展。深入开展全民健身活动，提高竞技体育水平，完成万人体育场改造任务，增加社区体育健身器材。

 建设“平安鹤岗”。完成武警扩编，建立特警队，实现110、120、119三台合一，健全社会治安防控体系，强化高密度、网络化的防控格局，严厉打击各类犯罪。

 强化安全生产。全面落实安全生产责任制，增强企业责任主体意识；增加安全生产投入，改善安全生产设施；加强安全生产监管，实行安全控制指标和“一票否决”制度。以“一通三防”为重点，抓好煤矿安全，杜绝3人以上重特大事故；针对薄弱环节和重大问题，加强消防、交通、危险化学品、非煤矿山、建筑、人员密集场所的安全和食品、药品安全专项整治，确保不发生重大事故。

 要抓好民族、宗教、外事、侨务、民政、双拥、人口、国防、人防、档案、司法、人事、物价、统计、地震、残联等各项社会事业发展。

 今年要筹资和投入1.5亿元，完成事关社会事业发展的10件大事。一是完成中小学校6000平方米D级危房改造；二是建设6000平方米市职教中心教学楼；三是建设市一中5000平方米图书馆和6000平方米体育馆；四是建设医疗废物处置中心；五是完善启用传染病院；六是改造市影剧院；七是建设广播电视演播大厅；八是改造万人体育场；九是改造煤矿安监设施，搞好煤矿安全专项整治；十是购置消防特种装备，改造消防二中队。

 （十二）切实关注民生民利，努力构建和谐社会。

 坚持以人为本、执政为民宗旨，更加关注民生、救助民困、谋求民利、造福民众，让全市人民共享改革发展成果，促进和谐社会建设。

 健全社会保障体系。认真做好社保试点后续管理服务工作；以非公有制经济从业人员、灵活就业人员和新建企业为重点，加快“五险”扩面和收缴；确保养老保险金、失业保险金和城市居民最低生活保障金按时足额发放。

 扩大就业和再就业。实现城镇新增就业2.2万人，城镇登记失业率控制在4.6%以内。扩大小额贷款规模，支持下岗失业人员自主创业；继续开发公益性就业岗位，扩大“4050”人员就业；更新就业观念，拓宽就业渠道，引导下岗失业人员在服务业、建筑业、煤炭生产、农业、畜牧业等多领域创业就业；巩固劳务输出品牌，做好对北京、上海、青岛、大连、广东等地的各类专业性劳务输出，建设具有专业特色的劳务输出基地。

 改善生产生活条件。既要抓好增强城市功能的供水、排水、道路、电力、通讯、供热、供气、城区防洪、医疗废物处置、沉陷区治理、棚户区改造等城市基础设施、公益福利设施大项目建设，又要高度重视并抓好事关市民日常生活的背街小巷的道路、路灯、排水、垃圾、公厕、物业、社区服务等薄弱环节建设，进一步方便百姓生活。落实国家关于公务员和事业单位工作人员工资改革各项政策，引导督促各类生产经营企业不断提高从业者的工资水平。全部免费开放城市公园。

 帮助扶持困难群体。健全社会救助体系，建立贫困学生助学资金、困难劳模扶贫资金和特困户扶贫解困救助资金，构筑政府引导、社会参与的救助保障体系；开展医疗、教育、住房、就业、救灾、法律援助等专项救助，推进城市扶贫帮困活动。整合农村敬老服务设施，实现集中办院，规划建设城市老年公寓小区；建设廉租房，扩大特教学校，启动低保医院、平价药店试点等公共扶贫救助事业。

 完善社区服务功能。将社会保障、宣传、文化、卫生、信访、社会治安、物业管理等职能延伸至社区。把社区建设纳入国民经济发展总体规划，列入各级政府重要日程；把社区经费和办公用房、社区干部工资列入市、区两级财政预算，加快社区基础设施建设，提高社区干部待遇。

 今年要筹资和投入近亿元，完成20件涉及改善民生、解决民困、维护民利的实事。一是加大城市防洪力度，基本解除居民区经常被淹状况；二是再免费开放两个城市公园，使市内公园全部免费开放；三是建设5000平方米廉租房；四是建设一所3000平方米的农村敬老院；五是完成5万平方米城市老年公寓小区规划及部分基础设施建设；六是建设5000平方米的老年活动中心；七是试办一所低保医院、一个平价药店；八是全面实施九年义务教育一费制和对困难学生“两免一补”政策；九是扩大并完善教育残疾儿童的特教学校；十是建成民政救助站；十一是基本解决市内社区办公服务用房；十二是提高社区干部补助标准，每人每月提高100元，并对每个社区每年安排3000——4000元经费；十三是新购买200个公益岗位，安排“4050”下岗人员，用于补充社区工作人员；十四是为社区新安装体育路径器材30套；十五是增加小额贷款担保资本金，用于下岗人员创业再就业；十六是提高低保补贴标准10%；十七是对未参保的集体企业退休人员，从3月1日起按低保标准发放生活费；十八是提高供暖补贴标准，由过去的补贴70%提高到90%；十九是建设殡葬服务中心；二十是健全街、路、门牌，实现入巷到户。

 从促进经济发展、推动社会进步、关注公众利益出发，采取政府引导、社会参与、多路并举的方式，加大筹资力度，保障重点工作。市政府安排1000万元，设立十项专项资金。建立优秀拔尖人才奖励资金，招商引资奖励资金，见义勇为奖励资金，纳税多、安置就业多、有突出贡献的企业家奖励资金，贫困学生助学资金，劳模解困资金，扶贫解困救助资金，特困户住房补贴资金，突发事件应急安置资金，新农村基础设施建设资金。以上十项资金由市政府分别制定专项管理使用办法。

 四、加强政府行政能力建设

 加快经济发展、构建和谐社会的时代要求和繁重任务，安全稳定的艰巨工作和重大责任，对政府工作提出了更高的要求。我们必须适应形势和发展需要，切实加强政府自身建设特别是行政能力建设，担负起时代和人民赋予的历史使命。

 （一）转变职能，切实增强依法行政的能力。

 进一步转变政府职能。根据经济社会发展需要，切实把政府职能转变到经济调节、市场监管、社会管理、公共服务上来。要更加注重用经济和政策手段宏观调控，促进发展；更加注重公共设施建设，强化公共服务和管理；更加注重培育、建设市场，加强市场监管；更加注重中介组织和社区等基层组织建设，推进社会民主管理、居民和村民自治管理，切实提高政府服务管理水平。

 大力实施依法行政。认真落实依法治国方略，坚持依法行政。进一步增强法制观念，自觉接受人大的法律监督、政协的民主监督和人民群众的社会监督、舆论监督，严格按照法定权限和程序行使权力、履行职责，做到依法办事、依法决策、依法处理问题。要加强体现依法行政的机制制度建设，把政府工作纳入法制化轨道。

 （二）强化学习，切实增强驾驭经济工作的能力。

要切实提高政府对经济工作的领导、管理能力。

　加强学习。政府组成人员要进一步增强经济工作中心地位的意识、发展第一要务的意识、政府对经济工作的责任意识，自觉以经济工作为己任，不论分管什么工作、任职什么部门，都要加强对经济理论和相关管理知识、专业知识的学习，特别是加强对市场经济相关理论知识的学习，努力成为经济工作的行家里手，提高对经济工作特别是市场经济的熟悉程度、管理服务能力。

 加强对市情和经济实践的研究。既要深入研究市场，适应市场需求，还要研究市情，找准比较优势和客观劣势。依托比较优势，培育经济优势和竞争优势，避免错误决策和投资劣势项目，扬长避短，规避风险，降低发展代价，减少损失。要深入研究市场经济条件下，政府宏观调控、服务推动经济发展的工作机制和社会管理机制，特别是要探索作为经济欠发达地区在市场经济体制不完善的现实情况下加快经济发展的途径和出路、政府推动经济工作的方式和方法，努力建立能够从根本上、全局上长期起作用的长效机制，卓有成效地加快经济发展。

 牢牢把握经济工作重点。在艰巨繁重的经济任务和纷繁复杂的困难、矛盾和实际问题面前，要善于把握重点、突破难点、解决问题、化解风险。我市的重点就是要解决资源加工、项目建设、招商引资、全民创业、发展环境和人才领导等问题，要抓住这些重点难点问题，组织攻坚，加以突破，推动经济又快又好发展。

 （三）勇于实践，切实增强解决实际问题的能力。

 要有效解决历史积淀和改革发展中遇到的困难、风险、矛盾和问题。

 要振奋精神，有良好的精神状态。要以对党和人民事业高度负责的强烈事业心、责任感，以忠于职守、敬业尽责的职业道德、积极务实的态度，勇敢面对工作中的困难、矛盾和问题，不回避矛盾，不畏惧困难，辛勤工作，科学处置。

要勇于承担风险，触及矛盾。政府工作确实经常面临一些棘手的问题和巨大的风险，但越是困难大、风险大的问题，解决得好，成效更大，拉动作用更大。政府工作中，解决问题、克服困难、消化风险的能力决定经济发展、事业进步的水平。政府工作人员必须主动深入实践、敢于碰硬，不怕困难和风险，肯下硬功夫，解决实际问题，不惜个人代价换来工作进步、经济发展和人民利益。

 要善于处理复杂局面，解决急难问题。当前经济工作中的环境制约问题，改革中一些单位的大锅饭体制机制问题，招商引资和项目建设中的政策问题、风险问题，稳定工作中的历史积淀的大量实际问题，企业改制中的遗留问题和信访问题，一些单位的内耗问题，以及计划经济体制下长期形成的一些体制机制性弊端、落后的思想观念，还有经常会出现的一些群体性事件、突发性问题，长期困扰政府工作，障碍经济发展。政府各级领导干部必须求真务实，真抓实干，潜心研究，精心解决，努力提高解决实际问题、应对处置突发事件的能力和效率，腾出更多的时间和精力抓发展、抓项目、抓招商、抓人民群众生产生活中的重大问题和事项。

（四）努力探索，切实增强构建和谐社会的能力。

 构建和谐社会是政府加强社会建设管理的崭新而重大的任务。政府组成人员必须与时俱进、创新务实，积极探索构建和谐社会的有效途径，提高构建和谐社会的行政能力。在加快发展的基础上，要重点解决好四个问题。

 一要注重使发展更多地惠及人民群众，更多地关注民生民利民困。以造福最广大人民群众为改革发展决策的出发点、落脚点，让人民群众在改革发展中更多地受益，更多地支持改革发展。

 二要更多注重社会公平。在社会主义初级阶段，作为经济欠发达地区，要坚持效率优先、加快发展，但同时必须注重社会公平，照顾到全体人民的利益，最大限度地调动、发挥、保护最广大人民群众的积极性、创造性，形成最强大的发展力量。因此要更好地实现经济社会的全面发展、各个方面的协调发展、各部分群体利益的统筹兼顾，防止片面性、极端化。

 三要更多地关注救助弱势群体、困难层面。关心帮助弱势困难群体，解决好生产生活问题，是建设和谐社会的重要任务、重要标志，也是重点难点。要落实以人为本、执政为民的宗旨，更好地解决他们的困难，让改革发展的成果更多地惠及到他们。

 四要以做好信访工作为重点，加强社会矛盾化解工作。政府工作人员要体察下情、民情，以高度负责、积极务实的态度，深入群众了解民情民意、民疾民苦，认真负责、积极有效地解决民诉民求问题，善于解惑释疑、活血化淤、调节纠纷、处理矛盾、实现稳定。

（五）解放思想，切实增强改革创新的能力。

 要勇于打破常规，改革创新，为加快发展注入强大生机和活力。

 进一步解放思想，注重思想理论能力的建设。思想决定行动，思路决定出路。不但在经济发展和工作上要向先进发达地区学习，更要在思想观念上向先进发达地区学习。坚持从实际出发，坚持改革创新，不断突破经济滞后而形成的落后观念，不断突破发展缓慢而产生的思想僵化，坚持邓小平理论和“三个代表”重要思想，以科学发展观为统领，在事关改革发展全局的重点问题上持续解放思想，做到与时俱进、勇于创新、善于打破常规，用新思路、新办法、新措施实现新突破、新进步、新发展。

 善于创新机制制度，建立完善长效机制。要牢固树立依法治市的思想，善于从治本的角度逐步将卓有成效的工作经验、工作措施机制化、制度化。去年，政府工作报告提出了十个方面的机制制度建设，对加强政府建设和推进工作起到了较好的作用，今年要继续坚持并加大落实力度，不断完善创新，切实提高政府工作和自身建设规范化、法制化水平。

 （六）严于律己，切实增强拒腐防变的能力。

 要切实搞好党风廉政建设和拒腐防变能力建设，全面落实党中央、省委、市委的各项部署，以严于律己、提高自身拒腐防变能力为重点，提高政府廉政建设水平。要树立正确的人生观、价值观、政绩观、权利观，健康向上，严格要求，防微杜渐，严守廉政防线，做到清正廉洁。要严格要求家人、亲友和身边工作人员，带头遵纪守法。

 同时，还要切实加强政府的工作作风建设，密切同人民群众的联系，加大力度，强化落实，全面提高法制政府、廉洁政府、阳光政府、高效政府、务实政府、有为政府、为民政府、服务政府、诚信政府的建设水平。

 各位代表，2006年是实施“十一五”规划的开局之年，是振兴东北老工业基地的关键一年，是我市抢抓机遇、乘势而上、快速发展至关重要的一年。历史的经验反复证实，我市近年来的发展实践更加雄辩的证明，发展是全市人民的根本利益、共同大局，让我们高举邓小平理论、“三个代表”重要思想伟大旗帜，以科学发展观为统领，坚持以经济建设为中心，在省委、省政府和市委的正确领导下，万众一心，合力攻坚，清正廉洁，真抓实干，咬住青山，坚持数年，努力实现“十一五”规划的宏伟目标和今年的各项任务，推动全市经济社会大发展、快发展、跨越发展、科学发展，造福全市人民，打牢后劲基础，增强长远竞争力，为实现我市经济社会全面、健康、持续、快速发展和构建平安和谐鹤岗而努力奋斗！
